# Telerehabilitation in Hip and Knee Arthroplasty: A Narrative Review of Clinical Outcomes, Patient-Reported Measures, and Implementation Challenges

**DOI:** 10.3390/jfmk10040370

**Published:** 2025-09-26

**Authors:** Rocco Maria Comodo, Daniele Grassa, Alessandro El Motassime, Guido Bocchino, Riccardo Totti, Andrea De Fazio, Cesare Meschini, Giacomo Capece, Giulio Maccauro, Raffaele Vitiello

**Affiliations:** 1 Orthopaedics and Trauma Surgery Unit, Catholic University of the Sacred Heart Rome, 20123 Milan, Italy; roccocomodo96@gmail.com (R.M.C.); da.grassa95@gmail.com (D.G.); alessandroelmotassime@gmail.com (A.E.M.); guido.bocchino@hotmail.it (G.B.); riccardo.totti20@gmail.com (R.T.); andrea.defazio01@icatt.it (A.D.F.); cesare.meschini01@icatt.it (C.M.); giulio.maccauro@unicatt.it (G.M.); lele.vitiello@gmail.com (R.V.); 2 Department of Ageing, Neurosciences, Head-Neck and Orthopaedics Sciences, Orthopaedics and 3 Trauma Surgery Unit, Fondazione Policlinico Universitario Agostino Gemelli IRCCS Rome, 00168 Rome, Italy; 3 Orthopedics and Trauma Surgery, Mater Olbia Hospital, 07026 Olbia, Italy; 4 U.O.C. Orthopaedics and Traumatology, Pellegrini Hospital, 80134 Naples, Italy

**Keywords:** telerehabilitation, total hip arthroplasty, total knee arthroplasty, PROMs, digital health, virtual reality, postoperative rehabilitation

## Abstract

**Background**: Total hip and knee arthroplasty are common procedures for end-stage osteoarthritis, with rehabilitation playing a central role in functional recovery. Conventional face-to-face programs are often limited by accessibility, costs, and logistical barriers. Digital telerehabilitation has been increasingly investigated as an alternative. This review aims to summarize current evidence on its effectiveness, patient-reported outcomes, satisfaction, and economic impact. **Materials and Methods**: A narrative review was conducted using Medline, Web of Science, and Scopus up to April 2025. Randomized controlled trials and longitudinal studies evaluating telerehabilitation after total hip or knee arthroplasty were included. Data were extracted on functional performance, pain, autonomy, quality of life, patient satisfaction, and cost-effectiveness. **Results**: Across multiple RCTs, telerehabilitation produced functional outcomes generally comparable to conventional rehabilitation, with some studies reporting superior short-term improvements. For example, in a retrospective trial, Timed Up and Go improved by −8.0 ± 2.6 s in the digital group versus −4.9 ± 2.5 s with standard care (*p* < 0.01). Tablet-assisted programs reduced Five Times Sit-to-Stand times to 11.7 s at 6 months compared with 14.7 s in controls (*p* = 0.05). In hip arthroplasty, digital rehabilitation resulted in higher active flexion (97.4° vs. 89.9°, *p* = 0.018) and abduction (51.7° vs. 43.8°, *p* = 0.024). Quality-of-life measures, such as EQ-5D VAS, also showed improvements (82.9 ± 4.3 vs. 68.7 ± 4.6 at 6 months). Some studies reported higher patient satisfaction, for instance, a VR-based RCT found GPE at day 15 of 4.76 ± 0.43 in the intervention group versus 3.96 ± 0.65 in controls (*p* < 0.001). **Conclusions**: Telerehabilitation after hip and knee arthroplasty appears to produce short-term functional and patient-reported outcomes comparable to conventional rehabilitation in selected populations. Evidence of superiority is limited and heterogeneous, and long-term effectiveness, equity, and cost-effectiveness remain uncertain. Heterogeneity in protocols and digital literacy barriers highlight the need for standardized guidelines and further independent validation.

## 1. Introduction

Osteoarthritis (OA) is a degenerative joint disease that primarily affects weight-bearing joints such as hips and knees, leading to pain, stiffness, reduced range of motion, and functional impairment [1]. Risk factors include age, obesity, joint injury, genetic predisposition, and mechanical stress [2]. Current management relies on a combination of non-pharmacological and pharmacological therapies aimed at symptom relief and functional improvement, such as physical therapy, weight management, NSAIDs, and intra-articular injections [3]. In end-stage OA, when conservative measures fail, total joint arthroplasty represents the gold standard treatment [4]. In recent decades, the number of hip and knee replacements has substantially increased worldwide, driven by the aging population and advances in surgical techniques [5,6].

However, the success of joint replacement depends not only on surgery but also on structured and effective postoperative rehabilitation. Rehabilitation is essential to restore joint function, recover muscle strength and mobility, and facilitate return to daily activities [7]. Yet, conventional face-to-face rehabilitation often encounters barriers: limited access in rural or underserved areas, long travel distances, high costs, and restricted availability of specialized professionals [8].

In this context, telerehabilitation has emerged as a feasible alternative to conventional programs. It allows remote assessment, supervision, and monitoring through technologies such as videoconferencing, mobile applications, web platforms, and wearable sensors, delivered synchronously or asynchronously, either fully remote or in hybrid models [9,10]. Reported advantages include improved accessibility, reduced travel burden, cost savings, and better adherence to home-based exercise programs. Recent systematic reviews and meta-analyses suggest that telerehabilitation is safe, effective, and potentially cost-efficient [11,12].

Nevertheless, evidence remains fragmented due to heterogeneity in technologies, outcome measures, and patient adherence. These gaps make it difficult to establish standardized protocols and generalize findings.

This narrative review aims to synthesize the current literature on telerehabilitation after total hip and knee arthroplasty, evaluating its effectiveness, safety, cost-efficiency, and patient-reported outcomes, and discussing how these findings relate to conventional rehabilitation, while highlighting both benefits and limitations.

## 2. Materials and Methods

This work was designed as a narrative review. A formal protocol was registered in PROSPERO (registration number 1150074), and the review process was conducted in accordance with PRISMA guidelines.

A comprehensive literature search was carried out up to April 2025 using Medline (via PubMed), Web of Science, and Scopus. The full electronic search strategy for PubMed was as follows: (“telerehabilitation” OR “tele-rehabilitation” OR “remote rehabilitation”) AND (“total hip arthroplasty” OR “hip replacement” OR “total knee arthroplasty” OR “knee replacement”). Equivalent search strings with adapted syntax were applied in Web of Science and Scopus. Only articles published in English were considered. In addition, the reference lists of the selected studies were manually screened to identify potentially relevant publications that were not retrieved during the initial database search.

Eligible studies included randomized controlled trials as well as prospective or retrospective longitudinal research. Studies were included regardless of blinding status, and adult patients of all types undergoing total hip or knee arthroplasty were eligible, without restrictions based on comorbidities or surgical indication. The target population consisted of adult patients undergoing total hip or knee arthroplasty. Interventions of interest were postoperative telerehabilitation programs delivered through mobile applications, web-based platforms, videoconferencing, wearable sensors, or virtual reality. Outcomes of relevance included clinical and functional recovery, patient-reported outcome measures (PROMs), cost-effectiveness, adherence, satisfaction, and complications. Systematic reviews and meta-analyses were excluded to avoid duplication of evidence, while conference abstracts were excluded because of insufficient methodological and outcome details for robust appraisal [Table 1].

The study selection process was performed in two steps. First, titles and abstracts were screened to remove studies that did not meet the eligibility criteria. Second, full-text articles were retrieved and assessed for final inclusion. Two reviewers independently conducted the selection and data extraction; disagreements were resolved by discussion with a senior author. Inter-rater reliability was quantified using Cohen’s kappa statistic to ensure consistency in study selection.

For each included study, the following information was extracted: study design, sample size, type of telerehabilitation intervention, comparator (if any), clinical and functional outcomes, patient-reported measures, complications, and implementation-related factors. Risk of bias was assessed independently by two reviewers using ROB-2 for randomized controlled trials and the Newcastle–Ottawa Scale (NOS) for observational studies. Discrepancies in scoring were resolved by consensus.

The extracted evidence was synthesized in pre-specified categories: (1) synchronous vs. asynchronous interventions, (2) hip vs. knee arthroplasty, and (3) study design (RCTs vs. cohort studies). This categorical approach ensured comparability across studies. Emphasis was placed on the level of clinical evidence, barriers to implementation, and potential economic implications.

**Figure 1:**
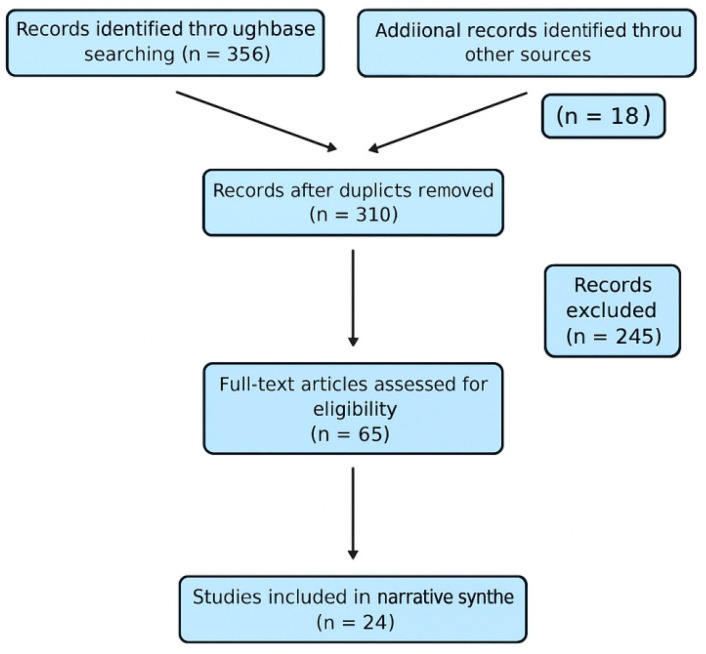
PRISMA Flow-diagram.

Although this review is not a systematic review with meta-analysis, a PRISMA-style flow diagram was developed to illustrate the study selection process, thereby increasing methodological transparency (Figure 1).

## 3. Results

### 3.1. Technologies and Modalities of Telerehabilitation

Telerehabilitation has gradually emerged as an alternative to traditional in-person rehabilitation, supported by digital technologies that enable remote interventions. Early implementations were based on synchronous modalities, such as videoconferencing and telephone calls, which allowed direct patient–therapist contact, immediate correction of exercises, and building of a therapeutic alliance. These sessions are particularly suitable for initial assessments or supervised training, but require a stable connection and simultaneous availability, limiting accessibility [13,14].

With the expansion of mobile networks, asynchronous modes have gained prominence. Mobile applications and web platforms enable patients to access exercise libraries, educational materials, reminders, and feedback in a flexible manner, often combined with progress tracking and periodic clinician input [15]. Wearable devices (accelerometers, inertial measurement units, gyroscopes) have been integrated to objectively monitor mobility parameters, while virtual reality systems provide immersive and motivating training environments [14]. Artificial intelligence has recently been applied to analyze biometric data and personalize rehabilitation pathways dynamically [16].

Some platforms, such as myMobility (Zimmer Biomet), have been investigated in industry-sponsored case series. They integrate educational material, PROMs collection (HOOS, KOOS, SF-36), and sensor-based activity tracking. When combined with robotic systems such as ROSA, preliminary studies report improved early adherence and functional recovery. However, no independent randomized controlled trial has yet validated this integration [17,18,19].

Other validated systems, such as StepApp, showed a strong correlation between remotely collected functional test data (6MWT, 10MWT, 30SST) and in-person measures, suggesting good reliability [20]. Nevertheless, adherence is influenced by patient-specific factors including caregiver support, digital literacy, and comorbidities [14,21].

### 3.2. Evidence of Effectiveness

The transition to telerehabilitation entails, at the same time, an evolution in the assessment of Patient-Reported Outcome Measures (PROMs): whereas in the past the focus was only on biomechanical parameters (e.g., pain, strength, range of motion), today increasing attention is being paid also to indicators such as patient satisfaction, self-efficacy, health-related quality of life (HRQoL) and cost–benefit analysis, reflecting a more holistic approach to evaluating rehabilitation effectiveness [14]. Patient-Reported Outcome Measures (PROMs) are standardised instruments used to collect information directly from patients about their perception of their health status, physical function, pain, quality of life and satisfaction with the treatment received. They are used in clinical and research settings to evaluate the effectiveness of therapeutic and rehabilitation interventions. PROMs can be categorised into(1) functional outcomes (e.g., TUG, 6MWT), (2) autonomy in daily activities (e.g., FIM, Barthel), (3) pain (e.g., VAS, NRS), (4) mental health and self-efficacy (e.g., HADS, SEMCD), (5) quality of life (e.g., EQ-5D, SF-36), (6) specific joint outcomes (e.g., HOOS, KOOS, WOMAC, HHS), and (7) perceived satisfaction (e.g., GPE).

PROMs have been used to capture both functional and patient-centered outcomes. To ensure consistency, we extracted and reported whenever possible the sample size, mean or median values, effect sizes, confidence intervals, and *p*-values. Results are presented in structured comparative tables, contrasting telerehabilitation with conventional rehabilitation for mobility, pain, quality of life, and satisfaction.

### 3.3. Physical Function and Motor Performance

Functional performance is a core domain for assessing rehabilitation effectiveness. The available studies indicate that telerehabilitation is at least equivalent, and in some cases superior, to conventional rehabilitation for motor outcomes. For example, in the retrospective trial by Venosa et al., patients in the telerehabilitation group improved their Timed Up and Go (TUG) score from 20.0 ± 2.0 to 12.0 ± 1.5 s (Δ −8.0 ± 2.6), while the control group improved from 18.0 ± 1.5 to 13.1 ± 2.0 s (Δ −4.9 ± 2.5), with a statistically significant difference (*p* < 0.01) [22,23,24]. Similarly, Wijnen et al. reported that patients using a tablet-based intervention achieved 11.7 s in the Five Times Sit-to-Stand Test (FTSST) at six months, compared to 14.7 and 14.0 s in control groups (*p* = 0.05) [25].

In StepApp validation studies, patients increased their 6-Minute Walk Test (6MWT) distance from 128.0 to 210.0 m, and their 10-Meter Walk Test (10MWT) speed from 0.37 to 0.40 m/s(slow) and 0.60 to 0.90 m/s(fast) (*p* < 0.0001) [26,27,28]. Lower limb strength and endurance also improved, as shown by the 30-Second Sit-to-Stand Test (30SST), where repetitions increased from 6.0 to 8.0 (*p* < 0.0001), and by Short Physical Performance Battery (SPPB) scores, which rose from 5.0 to 7.0 points (*p* < 0.0001).

In a randomized controlled trial by Correia et al., telerehabilitation following hip arthroplasty resulted in active flexion of 97.4° versus 89.9° in conventional rehabilitation (*p* = 0.018) and abduction of 51.7° versus 43.8° (*p* = 0.024) at six months. Peak torque measurements demonstrated a pronounced improvement in hip abductor strength (+69.6 Nm/kg×10 TR vs. +62.6 control; *p* = 0.028; Cohen’s d = 2.4) [29]. These results indicate that digitally monitored rehabilitation can achieve comparable or superior gains in mobility, strength, and joint function [Table 2].

### 3.4. Autonomy and Activities of Daily Living (ADL)

Recovery of independence in ADLs is a key goal after joint replacement. In the study by Cottrell et al., telerehabilitation patients achieved mean Functional Independence Measure (FIM) scores of 111.7 ± 3.1 at six months, significantly higher than 98.6 ± 5.1 in the control group (*p* < 0.001) [30,31,32]. The Barthel Index outcomes were similarly high in both telerehabilitation and conventional groups (98.6 ± 2.8 vs. 99.2 ± 1.3, *p* = ns) [33], indicating near-complete functional independence [Table 3].

### 3.5. Pain

Pain reduction was observed in both TR and conventional groups. In Venosa et al., telerehabilitation led to a decrease in rest pain from 2.2 ± 0.1 to 0.8 ± 0.2, compared to 1.7 ± 0.4 to 0.6 ± 0.1in controls (*p* < 0.01) [23]. Pain during movement improved similarly in both groups (Δ −2.0, *p* = 0.93). In Lippi et al., patients using StepApp reported a reduction from 2.0 to 1.0 (*p* = 0.042) [26]. Other instruments, such as WOMAC, HOOS, and KOOS pain subscales, showed trends favoring telerehabilitation, although some differences were not statistically significant [Table 4].

### 3.6. Psychological State and Self-Efficacy

Psychological well-being and self-efficacy are important determinants of adherence. The Self-Rating Anxiety Scale (SAS) scores decreased significantly in the telerehabilitation group from 42.4 ± 3.1 to 29.3 ± 1.6, compared to 46.2 ± 3.5 to 33.8 ± 2.6 in the conventional group (*p* < 0.001) [35,36,37,38]. Self-Efficacy for Managing Chronic Disease (SEMCD) scores increased moderately in both groups (5.2 → 7.3 TR vs. 5.1 → 6.8 control; *p* > 0.4) [39,40], and Self-Efficacy for Rehabilitation Outcome (SER) assessments indicated continuous improvement with higher adherence to digital platforms [41,42] [Table 5].

**Table 4:** Pain Outcomes.

Outcome	Study	n (TR/Control)	Baseline	Post-Rehab	Δ	*p*-Value
VAS Rest	Venosa et al. [23]	30/30	2.2 ± 0.1/1.7 ± 0.4	0.8 ± 0.2/0.6 ± 0.1	−1.4/−1.1	<0.01
VAS Movement	Venosa et al. [23]	30/30	4.5 ± 0.3/4.3 ± 0.4	2.5/2.3	−2.0/−2.0	0.93
NRS	Lippi et al. [26]	20	2.0	1.0	−1.0	0.042
WOMAC Pain	Salaffi et al. [36]	20/20	6.0 ± 4.5/6.3 ± 4.5	2.93 ± 4.55/6.27 ± 4.55	−3.07/−0.0	0.215

**Table 5:** Psychological Outcomes.

Outcome	Study	n (TR/Control)	Baseline	Post-Rehab	Δ	*p*-Value
SAS	Wang et al. [38]	25/25	42.4 ± 3.07/46.21 ± 3.53	29.26 ± 1.63/33.81 ± 2.62	−13.1/−12.4	<0.001
SEMCD (1–10)	Sun et al. [40]	28/27	5.2/5.1	7.3/6.8	+2.1/+1.7	0.40
SER	Wang et al. [42]	20	5.0	7.8	+2.8	–

### 3.7. Quality of Life (QoL)

HRQoL outcomes demonstrated favorable trends with telerehabilitation. In Lin et al., EQ-5D VAS scores at six months were82.9 ± 4.3 in the TR group versus 68.7 ± 4.6 in controls, with a large effect size but a non-significant *p*-value (0.093) [43,44,45]. SF-36 physical function scores in Wijnen et al. increased from 61.4 ± 20.2 to 89.0 ± 10.6 in TR vs. 62.1 ± 18.9 to 74.6 ± 15.8 in controls; *p* = 0.07; Cohen’s d = 1.5 [25]. HOOS/KOOS QoL scores were similar between groups, indicating high overall quality of life irrespective of delivery mode [46,47,48,49] [Table 6].

### 3.8. Functionality

Joint-specific PROMs consistently demonstrated equal or superior outcomes for telerehabilitation. Wu et al. reported significantly higher Harris Hip Scores at 1, 3, and 6 months in TR patients (84.2 ± 3.1 vs. 77.3 ± 4.9 at 6 months, *p* < 0.001) [50,51]. Similarly, HOOS sport and quality-of-life subdomains improved more in TR groups compared to conventional care [25], while KOOS and Oxford Knee Scores reflected comparable or slightly better gains with digital interventions [52,53,54] [Table 7].

### 3.9. Satisfaction and Subjective Perception

Patient satisfaction was generally higher with telerehabilitation. In Fascio et al., Global Perceived Effect (GPE) scores on postoperative day 15 were 4.76 ± 0.43 in TR vs. 3.96 ± 0.65 in controls (*p* < 0.001) [34]. Rossi et al. reported that approximately 85% of telerehabilitation users rated their experience as “satisfactory” or “very satisfactory,” with more than 70% preferring a digitally supported approach [19].

### 3.10. Proprioception and Digital Engagement

Proprioceptive outcomes are less frequently reported but show measurable benefits with VR-assisted rehabilitation. Gianola et al. demonstrated that the proprioceptive similarity index increased from 47.3% to 62.8% in the VRRS group, compared to a marginal improvement of 3.2% in controls (*p* = 0.002) [55,56,57,58], indicating enhanced joint position sense and movement accuracy with immersive digital interventions.

### 3.11. Economic and Accessibility Aspects

Telerehabilitation has been associated with reduced direct and indirect healthcare costs. Nelson et al. demonstrated cost-effectiveness of home-based TR compared to inpatient care post-THA [55]. GaitSmart, a sensorized system integrated with digital coaching, generated an average saving of £450.56 per patient and an increase of 0.02 QALYs, establishing a dominant strategy [54]. A meta-analysis of 14 studies showed a 90% probability of cost-effectiveness at a willingness-to-pay threshold of USD 30,000/QALY, with estimated savings ranging from USD 1200 to USD 6500 per patient [55]. Nonetheless, infrastructural and socio-economic barriers, such as limited internet connectivity or low digital literacy, remain critical challenges, especially in rural or disadvantaged regions [49].

## 4. Discussion

Telerehabilitation has been increasingly applied in healthcare, offering an alternative to conventional in-person therapy, although its effectiveness remains context-dependent and influenced by multiple factors.

Despite its potential advantages, telerehabilitation presents technological barriers that may compromise treatment, especially in the elderly population. Among the most frequent problems identified in the literature are unfamiliarity with digital devices, the need for stable, high-speed connections, and difficulties in configuring and maintaining systems, as reported by Chen et al. [22]. Many users, especially those with physical or cognitive disabilities, may face significant obstacles in using keyboards, software or video devices, aggravated by a lack of technical support and non-intuitive interfaces [15].

Further critical issues concern the need for software updates, the management of multiple peripheral devices (cameras, sensors, remote controls), and the intervention of clinical staff not always trained in technology, which may slow down or compromise patient interaction. Gianola et al. [58] highlighted that sensory and cognitive problems typical of older individuals, such as visual impairment or memory difficulties, necessitate a simplified and inclusive design of user interfaces.

The implementation of tele-neurorehabilitation is also hindered by practical problems such as uneven access to smart devices, network interruptions, poor lighting, and difficulties in receiving timely technical assistance, with a significant impact on acceptance and perceived effectiveness, especially among elderly patients [19]. These technological and infrastructural barriers may also introduce selection bias, as patients willing and able to use digital platforms likely differ systematically from those who cannot.

Telerehabilitation can serve as a resource for patients living in peripheral or underserved areas, often affected by poor healthcare facilities, economic constraints, and limited mobility [52]. Several studies indicate that it enables better allocation of healthcare personnel, ensures continuity of care, and reduces systemic costs, especially in post-pandemic contexts [51,52]. However, publication bias may overestimate these benefits, as negative trials and failed implementations are less likely to be reported. Some cited studies are also industry-sponsored, raising potential conflict-of-interest concerns.

The adherence of elderly patients is a crucial dimension for clinical effectiveness. Recent studies show that most patients can follow the programme at a distance, but hindering factors related to age, educational level, comorbidities, and family support remain [14]. Patients who are more digitally literate, supported by family, or with higher socioeconomic status are more likely to adhere, highlighting disparities in access and outcomes.

Wijnen et al. [26] observed good adherence in moderately elderly patients (up to 75 years) to a programme supported by tablet and telephone coaching, but emphasized the need for initial onboarding. Weekly exercise frequency (more than 5 days) and participation in telephone coaching contributed to higher clinical improvement than controls. However, Chen et al. [22] highlight that barriers remain in patients over 65, including lack of understanding of the treatment pathway, fears related to pain or complications, and logistical difficulties. These factors often result in reduced rehabilitation efforts. Such heterogeneity underscores the need for caution in generalizing findings across populations.

Finally, the use of mobile apps and social messaging systems (e.g., WeChat) was perceived positively in terms of accessibility, but digital literacy remains a limitation for some elderly people, especially in the absence of personalised technical guidance [56]. Socioeconomic disparities, limited digital competence, and infrastructural gaps continue to challenge the full implementation of telerehabilitation.

Currently, there is no universally accepted protocol for the administration of telerehabilitation programmes, limiting replicability, comparability of outcomes, and integration into healthcare systems [15]. Variability in duration, intensity, content, and mode of administration complicates establishing clear therapeutic thresholds and hinders evidence synthesis.

Ethical and legal considerations require more extensive analysis. The protection of personal data, confidentiality, and cybersecurity are critical, particularly in cross-border contexts where data governance laws vary [19]. Algorithmic opacity in AI-based platforms may lead to systematic errors, raising questions of liability in case of harm during unsupervised exercise [20]. Clinicians and developers face unclear responsibilities, and patients may be exposed to risks without robust legal safeguards. Transparent reporting, regulatory oversight, and standardized ethical frameworks are necessary to mitigate these risks.

## 5. Conclusions

Telerehabilitation represents a promising modality for delivering post-arthroplasty rehabilitation, with evidence suggesting non-inferior short-term outcomes in selected patient cohorts compared to conventional face-to-face rehabilitation. It may promote joint mobility, muscle strength, and pain control while also considering patient-reported outcomes such as satisfaction, adherence, and health-related quality of life. The integration of digital tools, including virtual reality, smart wearable systems, mobile applications, and AI platforms, allows for customization, monitoring, and patient engagement. However, the current literature is heterogeneous, and evidence on long-term effectiveness, equity of access, and real-world cost-effectiveness, particularly in elderly populations, is limited.

Moreover, most studies originate from a few countries with established telerehabilitation infrastructure, limiting the generalizability of findings to other healthcare settings and populations.Although telerehabilitation has the potential to reduce transport costs, lost working time, and the need for therapists’ physical presence, these economic benefits remain context-dependent and are not fully established across diverse healthcare settings. Future research should focus on developing evidence-based clinical practice guidelines, refining remote assessment tools, and conducting robust trials to evaluate long-term outcomes. Furthermore, ethical and legal issues, including privacy, clinician accountability, and algorithmic transparency, must be addressed to ensure safe and equitable implementation. In summary, telerehabilitation is a promising and adaptable approach, often non-inferior in the short term, but further high-quality studies and standardized protocols are urgently needed before definitive claims about its effectiveness and sustainability can be made.

## Figures and Tables

**Table 1:** Inclusion and Exclusion Criteria.

Inclusion Criteria	Exclusion Criteria
Randomized controlled trials, prospective or retrospective longitudinal studies	Case reports, expert opinions, editorials, letters, systematic reviews or meta-analyses, conference abstracts without full data.
Adult patients undergoing total hip or knee arthroplasty	Studies not directly addressing telerehabilitation after joint arthroplasty.

**Table 2:** Physical Function and Motor Performance Outcomes.

Outcome	Study	n (TR/Control)	Baseline (TR/Control)	Post-Rehab (TR/Control)	Δ (TR/Control)	*p*-Value
TUG (s)	Venosa et al. [23]	30/30	20.0 ± 2.0/18.0 ± 1.5	12.0 ± 1.5/13.1 ± 2.0	−8.0 ± 2.6/−4.9 ± 2.5	<0.01
FTSST (s)	Wijnen et al. [25]	25/25	14.8 ± 2.1/14.3 ± 1.8	11.7/14.7/14.0	−3.1/−0.3	0.05
6MWT (m)	Lippi et al. [26]	20	128.0	210.0	+82.0	–
10MWT Slow (m/s)	Lippi et al. [26]	20	0.37	0.40	+0.03	<0.0001
10MWT Fast (m/s)	Lippi et al. [26]	20	0.60	0.90	+0.30	<0.0001
30SST (reps)	Lippi et al. [26]	20	6.0	8.0	+2.0	<0.0001
SPPB (0–12)	Lippi et al. [26]	20	5.0	7.0	+2.0	<0.0001
Hip ROM Flexion (°)	Correia et al. [29]	28/27	87.0/85.0	97.4/89.9	+10.4/+4.9	0.018
Hip Abduction (°)	Correia et al. [29]	28/27	44.0/43.5	51.7/43.8	+7.7/+0.3	0.024
Peak Torque (Nm/kg × 10)	Correia et al. [29]	28/27	62.0/61.5	69.6/62.6	+7.6/+1.1	0.028

**Table 3:** Autonomy and Activities of Daily Living (ADL).

Outcome	Study	n (TR/Control)	Post-Rehab (TR/Control)	*p*-Value
FIM (0–126)	Cottrell et al. [33]	30/30	111.7 ± 3.13/98.64 ± 5.12	<0.001
Barthel Index (0–100)	Fascio et al. [34]	25/25	98.6 ± 2.8/99.2 ± 1.3	ns

**Table 6:** Health-Related Quality of Life.

Outcome	Study	n (TR/Control)	Baseline	Post-Rehab	Δ	*p*-Value
**EQ-5D VAS**	Lin et al. [45]	20/20	68.7 ± 4.6/68.7 ± 4.6	82.9 ± 4.3/68.7 ± 4.6	+14.2/0	0.093
**SF-36 Physical**	Wijnen et al. [25]	25/25	61.4 ± 20.2/62.1 ± 18.9	89.0 ± 10.6/74.6 ± 15.8	+27.6/+12.5	0.07
**HOOS QoL**	Zhou et al. [50]	30/30	68.7/68.7	68.7/68.7	0	0.70

**Table 7:** Joint-Specific Functionality.

Outcome	Study	n (TR/Control)	Baseline	Post-Rehab	Δ	*p*-Value
HHS (0–100)	Wu et al. [52]	30/30	66.35 ± 4.63/63.48 ± 4.49	84.23 ± 3.13/77.29 ± 4.95	+17.88/+13.81	<0.001
HOOS Sport	Wijnen et al. [25]	25/25	55.2/56.0	63.9/55.2	+8.7/−0.8	<0.05
OKS	2023 Study [53]	20/20	22 ± 1.3/23 ± 2.1	36 ± 2.7/35.1 ± 4.2	+14/+12	<0.01

## Data Availability

All the data we analysed and tables we compiled are available for any clarification.
